# Medical News and Miscellany

**Published:** 1899-10

**Authors:** 


### Medical News and Miscellany.

**Dr. J. S. Woods,** of Burkeville, died in that place August 25, 1899.

**Dr. C. H. Wilkinson** has been appointed City Physician of Galveston.

**Say!** In writing to advertisers don't forget to mention the "Red-Back." Be sure to read the ads.; they'll interest all "up-to-date" doctors.

**Erratum.**—In our last we said Dr. Sterling Price had removed from Richland to Powell, Tex. It should have been Dr. Jno. W. Price; Dr. Sterling Price is still at Barry, Tex.

**Gone**—Her Father (from the head of the stairs): "Ethel, is that young man gone?"

Ethel (in an ecstatic whisper): "Awfully, papa."—*Tid-Bits.*

**Dr. M. D. Knox,** of Hillsboro, Tex., died at that place August 27, 1899. Dr. Knox was a very eminent physician, and one of the oldest and most useful members of the State Medical Association.

**Dr. H. O. Sappington,** of Austin, Texas, a graduate of the Texas Medical College, has accepted a position as assistant physician to the mining company at Hartshorne, I. T., and has removed to that place.

**Professional Sarcasm.**—Young Doctor: "Congratulate me, old man, I'm just preparing to visit my first patient."

Young Lawyer: "Good, I'll go with you. Perhaps he has't made his will.—*Chicago News.*

**Dr. S. E. Hudson,** our "Junior," and Mrs. Hudson are spending some weeks at Chicago; the Doctor attending the Polyclinics and hospitals. He attended the meeting of the Mississippi Valley Medical Association in Chicago October 3 to 6. Dr. M. M. Smith, of the *Texas Medical News*, also attended the meeting, and has just returned, reporting having had a fine time.

**Marriage of an Austin Lady Physician.**—The JOURNAL received the following: William K. Cummings, M. D., Fanny E. Leak, M. D., married, Sunday evening, September 17th, 1899. Residence, after October 1st, Seattle, Washington.

---

**The New Orleans Polyclinic,** thirteenth annual session, opens November 20, 1899; closes May 10, 1900. Every inducement in clinical facilities for those attending. The specialties are fully taught. Further information, New Orleans Polyclinic, New Orleans, La.

---

**Change of Address.**—Dr. F. A. York has returned from Denver, Col., and located at Trinity, Tex. Dr. L. B. Roebuck has removed from Italy, Tex., to Rockdale, Tex.; Dr. W. R. Pfeuffer from Rockdale to New Braunfels; Dr. J. W. McDonald from Dripping Springs to Goforth; Dr. J. A. B. Liddell from Margueze to Middleton.

---

**A sea horse** is a sea horse
When you see him in the sea,
But when you see him in the bay
A bay horse then is he;
But the serum of a tainted horse
Is sero-therapy.—*Exchange.*

---

**A general** order for the vaccination of the employes of the Pennsylvania system has been issued by the company. It is far-reaching in its scope, as it takes in more than 100,000 men, besides their families. The new order is said to be the result of the present epidemic of smallpox in Pittsburg. A house-to-house canvass of the homes of the employes will be made.—*Louisville Medical Monthly.*

---

**David Harum** says the "golden rule" ought to read: "Do unto the other feller what he'd like to do to you, but *you* do it first." My version is: All you fellers who are in arrears for subscription (and a bill has been sent to every one, with a polite note), do unto the Red-Back as you would like for your delinquents to do unto you—pay up. My private opinion is that a debt for subscription is as much a matter of honor as a doctor's bill; and all doctors agree that a doctor's bill should be paid before anything else.

**Baths in Typhoid.**—The good effects of baths in typhoid are: (1) The reduction of the fever. (2) The intellect becomes clearer, the stupor lessens and the muscular twitchings disappear. (3) A general tonic action, particularly on the heart. (4) Insomnia is lessened, the patient usually falling asleep for two or three hours after each bath. (5) Most important of all, the mortality is, under this plan of treatment, reduced to a minimum.—*Osler, Exchange.*

---

**We call attention** to the professional card of Drs. Thompson and Saunders, Fort Worth, Tex. These eminent physicians limit their practice exclusively to diseases of women and to consultations. Dr. Saunders is dean of the medical school at Fort Worth; was recently president of the State Medical Association, and Dr. Thompson is Medical Director for the Equitable Life Insurance Company for Texas and the Indian Territory. They are both well and widely known as skillful and successful surgeons.

---

**Presents to Jefferson Medical College.**—J. Ackerman Coles, M. D., of Newark, N. J., has presented to Jefferson Medical College a large marble bust of Harvey, which was made in Rome in 1869, by Horatio Stone. Also the "Works of William Harvey, M. D., Physician to the King, Professor of Anatomy and Surgery to the College of Physicians," translated from the Latin, with a "Life of the Author," by Robert Willis, M. D., printed for the Syndenham Society, London, 1847. To these gifts Dr. Coles adds a bronze life-size medallion portrait of his father, Abraham Coles, deceased, and a life-size bronze copy of the celebrated Roman antique statute of the "boy extracting a thorn from the sole of his foot." These valuable gifts are to commemorate the 75th anniversary of Jefferson Medical College. Dr. Coles, Sr., was an alumnus of Jefferson, class of 1838.

---

**Say What You Mean.**—A Harmless "Line." Dr. Foscue, the Waco editor of our sprightly contemporary, *The Texas Medical News*, and *quandom* member of the medical board who examined Texas doctors for appointment in the Texas contingent of troops for the Cuban war, gives *The Medical News* for September an account of the examinations, etc., and a list of questions asked by the board. Among others is: "A line drawn from an inch below the umbilicus to a (stated) point near the spinal column, would injure what organs?" Well, a "line" will not injure *any* organ. We'd have thought it a "catch question" but for the fact that the doctor distinctly stated that the "questions were of a practical nature."

Here's another: A Young Husband. Our distinguished and classical friend of the *Medical Fortnightly*, in speaking of "Mrs. Eddy," says: "She had one child by the first husband, from whom she separated when he was four years old."

---

### To the Alumni, University of Texas.

---

AUSTIN, TEXAS, Sept. 8, 1899.

The following call for a meeting of the Alumni Association of the University of Texas, to convene at Dallas on October 21, has been issued and explains itself:

*To All Members of the Alumni Association of the University of Texas.*

October 21 has been set apart by the management of the Dallas State Fair as "University Day." The local alumni and ex-students of the University at Dallas have arranged for a reunion, banquet, foot ball game, and general celebration of the occasion, that will prove of interest to every ex-student and alumnus of the University who attends. The names of the Dallas alumni who have embarked on this enterprise, and the energy and enthusiasm with which they are forwarding the plan, constitute satisfactory guarantee of the success of "University Day," at the Fair.

A general meeting of the members of the Alumni Association of the University of Texas is hereby called to convene at Dallas, Texas, on October 21, 1899, for the purpose of consulting together on matters of interest and importance to the Association and to the University, and participating in the reunion and festivities of ex-students and alumni on that occasion.

Every member of the Association who can attend the meeting above called is requested to do so. The arrangements already made assure not only the success of the celebration, but also that those who attend will be able to take advantage of extremely low passenger rates on the railroads.

(Signed) V. L. BROOKS,
President Alumni Association.

### PRESS NOTICES.

The announcement that a book will soon be issued by the inimitable Dr. F. E. Daniel, editor of the *Texas Medical Journal*, at Austin, Texas, is creating a great amount of interest in the medical profession of the United States.

Dr. Daniel has been throughout life a keen observer, and possessed of marked ability in the direction of seeing things that escape the average man. In the conduct of his Texas Medical Journal, he has been a fiery, as distinct and definite as the color of the cover of the Journal which he is editing.

Anything, the product of his pen, is likely to resemble the aforesaid color of his red backed journal in that it will be "thoroughly read." Dr. Daniel is a veteran in the service as a practitioner and medical editor, and he was a surgeon in the Confederate Army during the Civil War, and as such, his experiences were unquestionably rich, rare and racy.

The book which he proposes to present to the profession will be known as "The Recollections of a Rebel Surgeon." That it will be written in his usual terse and trenchant style goes without saying. The MEDICAL MIRROR hopes that every doctor in the United States and everywhere else will buy a copy. The journalistic guild, medical, of the United States should see to it that such be the case.

Dr. Daniel as editor of *Daniel's Texas Medical Journal* (The "Red-Back" as it is affectionately called by its friends who form the bulk of the profession in Texas and adjoining States) has done some of the best, brightest and most original work done by any member of the editorial guild medical. There may come a time when he may be dead, but dull—never.—*Medical Mirror*, St. Louis.

---

To anyone familiar with the doctor's good qualities as a story-teller, the mere announcement of the appearance of the book will be sufficient; to others it may be said: it will be a "daisy." It is unnecessary to predict that this book will have a tremendous sale among physicians of the South; and doubtless many hundreds of copies will find their way into the North—for Dr. Daniel's reputation as a writer is as wide as the land, and no medical editor will be found who will not speak a good word for the book when issued.—*American Journal Surgery and Gynecology*, St. Louis.

---

Dr. Daniel's sense of humor is certainly sharp as a lance, and fairly twinkles with brightness; and his manner of making men and things the butt of ridicule, at will, is well-nigh inimitable.

Now, since he openly joins the professional ranks of mirth-provoking authors, in offering this book, his already good and wide reputation as a refreshing medical writer will stand him well in hand, insuring thousands of intelligent readers who, after being shaken up with his fun, as if by sudden bursts of hot and cold Turkish spray, will criticise his work, of course, most favorably.

We advise our subscribers to read it—evenings—after loosening waistcoat and collar buttons.—*Medical Progress*, March, '98, Louisville, Ky.

---

From our acquaintance with other works of the doctor's bright and incisive pen, we venture to predict that there will not be a dull page in the book. Look out for it, doctor; then get it and read it.—*Medical and Surgical Journal*, Atlanta, Ga., March, '98.

---

It is written in the doctor's inimitable style, and will make anyone grow fat who reads it. Dr. Daniel himself is not a fat man, as everybody knows who has seen him in his natural state, but he has the framework, and hopes to take on financial dropsy from the large sale of his work. You should keep a lookout for the book, which is expected to leave the press about November 15th.—*Texas Medical News.*

---

FUN AHEAD.—F. E. Daniel, M. D., of Texas, has written a book of his personal observation and experience as a rebel surgeon. Few men can write as Daniel can, and this book means a treat for every one who wishes to laugh and grow fat. It is, moreover, a matter of some concern that he should sell a copy to almost everybody, for there are strong hopes that the financial comfort of "Betty and the baby" will be materially helped by the revenue from this literary work. We say to all; buy his book, for both reader and publisher will be the gainers.—*Southern Clinic*, Richmond, Va., June, '98.

CONTENTS.

Introductory.
The Old Doctor Talks: His Retroscope.
Sunshine Soldiering.
Disinterested Solicitude: "A Fellow-feeling Makes us Wond'rous Kind."
The Doctor Gets Dinner.
How the Big Dog Went.
Bill and the Bumble-bee's Nest.
The Doctor Takes Supper With One of the F. F. V's.
The Doctor Routs the Federal Army.
A Violent Eruption of "Lorena."
Crossing the Cumberland.
An Extensive Acquaintance: "How Are You, Dickey?"
A Brush With the Seminary Girls.
The Doctor Takes Breakfast With the Yankees.
Perryville: The Doctor Scents the Battle From Afar.
Questionable "Spoils."
Recollections of Bacon (Likewise, of Pork).
Somebody's Darling.
A "Small Game," and a Big Stake.
The Little Captain's Toast, and What Happened.
Bushwhackers After the Doctor.
A Frog Story.
Poking Fun at the Medical Director.
Dr. Dick Taylor, of Memphis.
Presumptive Evidence.
A Close Call: A Bad Run, and a Worse Stand.
The Doctor Smuggles Contraband Supplies.
The Hospital Soldier.
The Hospital Dietary.
A "Medical" High-Daddy.
His Idea of Happiness.
Why He Was Weary.
Hospital Experiences.
Enchanted and Disenchanted.
A Friend In Durance Vile.
A Lookout Mountain Sprite.
The Clever Quartermaster: A Romance of Army Life in Chattanooga.
Love's Stratagem—The Doctor Puts Up a Job on the Major.
What Puzzled the Doctor.
Story of a Stump.
When the Dogwoods Were in Bloom: A Fish Story With Trimmin's.
Confederate States Shot Factory: ("Limited.") (Very.)
Dr. Yandell and the Turkey.
Old Sister Nick: Piety and Pies.
Wisdom in a Multitude of Counsel. (Nit.)
A Night at Meridian.
A Chapter for Doctors Only.
In the Land of the Blue Dog.
Jimmie Was All Right.
Circumstances Alter Cases: Any Port in a Storm.
Uncle Hardy Mullins: The Ways of Providence.
The Little Hu-gag, and the Great American Phil-li-lieu.
The Doctor Sees a Lady Home.
Fine Points in Diagnosis.
One on Thompson.
Halcyon Days.
The Doctor's Lament. (To His Lady Love.)
The Doctor Seeks Comfort in the Bible: What He Found.

Published by the Von Boeckmann, Schutze & Co. Publishing House, Austin, Texas. Orders should be addressed to Dr. F. E. Daniel, TEXAS MEDICAL JOURNAL, Austin, Texas.

**Leucorrhea.**—

℞ Acidi tannici ..... ℥ vj

Glycerin ..... ℥ xvj

M. Sig. One-half ounce to one pint of tepid water. Inject for five minutes into the vagina night and morning.

℞ Cupri sulphatis,
Zinci sulphatis,
Alum sulph ..... aa ʒ iss
Glycerini ..... ℥ vj

M. Sig. Injection.

℞ Acidi borac ..... ʒ j

Aq., tepid ..... Oj

M. Sig. Injection.—*Louisville Medical Journal.*

### A Fair Within a Fair.

The management of the San Antonio International Fair, which opens October 28th and closes November 8th, 1899, has engaged Prof. H. T. Atwater's grand museum exhibit at the coming Fair as one of the main attractions. The space occupied by this exhibit will be the entire south end of the gallery in the Main Exhibition Hall. For years Prof. Atwater has been adding to his collection of rare natural history specimens and curiosities from all parts of the earth, and in selecting specimens for an exhibit on an occasion of this kind he has a very valuable and extensive collection to draw from. The display will comprise two parts, one division of which will consist of natural history specimens, curios and relics from foreign lands, including some very curious animals, many beautiful birds of brilliant plumage, gorgeous butterflies and strange reptiles and insects. The other part of the exhibit will consist entirely of Texas specimens and this division will include a splendid natural history collection, some very rare mammals and birds, sea monsters, fish from the Texas coast and a large collection of relics and curiosities as well as numerous samples of Texas minerals and other commercial products from all parts of this State.

The sheepmen from the West will find samples of wool from the most celebrated flocks in England and other parts of the world. The cotton grower will see specimens of cotton from all parts of the earth. Those interested in mining will find samples of gold, silver and copper ore from the richest mines in the world. Students of natural history, or those scientifically inclined will find a vast amount of material that will require hours to inspect. This exhibit will be the headquarters of hunters and curio collectors and here they will tell the big fish stories and snake yarns. The sportsman from the North will be delighted with the fish and game display, and among other things he will see a thirty pound wild Texas gobbler in all the glory of its magnificent spring plumage.

Through the liberality of the Fair management, who are paying Prof. Atwater a large sum to make this display, the exhibit will be free to all visitors. This will no doubt be appreciated by all who attend, for at most of the modern exhibits it is the rule now-a-days to charge an extra admission fee to features of this kind.

